# Antarctic food web architecture under varying dynamics of sea ice cover

**DOI:** 10.1038/s41598-019-48245-7

**Published:** 2019-08-28

**Authors:** Loreto Rossi, Simona Sporta Caputi, Edoardo Calizza, Giulio Careddu, Marco Oliverio, Stefano Schiaparelli, Maria Letizia Costantini

**Affiliations:** 1 CoNISMa-Consorzio Nazionale Interuniversitario per le Scienze del Mare, Piazzale Flaminio 9, Rome, Italy; 2 Department of Environmental Biology, Sapienza University of Rome, Via dei Sardi 70, Rome, Italy; 3 Department of Biology and Biotechnologies “Charles Darwin”, Sapienza University of Rome, Zoology–Viale dell’Università 32, Rome, Italy; 4 Department of Earth, Environmental and Life Sciences (DISTAV), University of Genoa, Corso Europa 26, Genoa, Italy; 5 Italian National Antarctic Museum (Section of Genoa), University of Genoa, Viale Benedetto XV 5, Genoa, Italy

**Keywords:** Climate-change ecology, Ecological networks, Food webs

## Abstract

In the Ross Sea, biodiversity organisation is strongly influenced by sea-ice cover, which is characterised by marked spatio-temporal variations. Expected changes in seasonal sea-ice dynamics will be reflected in food web architecture, providing a unique opportunity to study effects of climate change. Based on individual stable isotope analyses and the high taxonomic resolution of sampled specimens, we described benthic food webs in contrasting conditions of seasonal sea-ice persistence (early vs. late sea-ice break up) in medium-depth waters in Terra Nova Bay (Ross Sea). The architecture of biodiversity was reshaped by the pulsed input of sympagic food sources following sea-ice break up, with food web simplification, decreased intraguild predation, potential disturbance propagation and increased vulnerability to biodiversity loss. Following our approach, it was possible to describe in unprecedented detail the complex structure of biodiverse communities, emphasising the role of sympagic inputs, regulated by sea-ice dynamics, in structuring Antarctic medium-depth benthic food webs.

## Introduction

The Ross Sea (Antarctica), the largest marine protected area in the world^1^, is considered a pristine ecosystem and an important biodiversity hotspot^2^. It is also a critical climate-change reference area, and a climate refugium for ice-dependent species. The protection of this area is thus essential for ethical reasons and because of its environmental and scientific value.

In the Ross Sea, biodiversity is strongly influenced by temperature and the consequent extent of sea-ice cover^3–5^, which is characterised by marked spatio-temporal variations^5–7^. The expected changes in seasonal sea-ice dynamics will be reflected in the structure of food webs, providing a unique opportunity to study the effects of climate change on biodiversity organisation^4,5,7,8^. However, food web complexity in polar ecosystems, along with undersampling, has limited efforts to predict future modifications due to climate change^9^. Specifically, the lack of scientific information on Antarctic communities and the ecological roles of their component species^10^ makes it difficult to predict how changes will affect mechanisms regulating key ecological processes, such as productivity and species interaction, and the associated community stability and vulnerability to species loss^11–13^. Understanding biodiversity architecture is thus necessary for a proper mechanism-based approach to the management and conservation of Antarctic coastal ecosystems under a climate change scenario.

One major factor impeding the reconstruction of Antarctic food web structure is the difficulty of correctly depicting the taxonomic basis of Antarctic biodiversity. Recent integrative approaches have revealed unexpectedly high levels of endemic and cryptic species, most of which have not yet been described, making classification of Antarctic biodiversity challenging. For this reason, as well as for the high degree of omnivory, the trophic role of many species remains uncertain or completely unknown^14^. In this context, stable isotope analysis (SIA) can provide clear information, integrated in space and time, about the trophic relationships that are established between organisms^5,15,16^ and can therefore be used to develop models of trophic structures^13,17,18^. With respect to other techniques of food web reconstruction, such as feeding experiments and stomach contents analysis, the stable isotope method has several advantages because, depending on the tissue analysed, it reflects the resources really assimilated by organisms in the short, medium and long term^7,15,18^. This can provide information on key food web characteristics such as the distribution of organisms and feeding links across trophic levels, and energy flows and matter circulation in the food web^5,13,15,17,18^ in a dynamic framework. However, due to trophic generalism and omnivory in populations, the currently used analytical methods can be ineffective. Since energy flows and nutrient transfer depend firstly on the individual foraging choices of organisms within the community, the concept of “trophospecies” (sensu Cohen and Briand^19^) can be adopted as part of a new mechanistic approach to determining trophic interactions in complex environments such as Antarctica. Here, based on the isotopic signatures of single individuals in the web, we introduce the concept of isotopic trophospecies (i.e. a group of individuals that share a similar position in the trophochemical bidimensional graph and the food web), reported as an Isotopic-Trophic-Unit (hereinafter: ITU).

**Figure 1:**
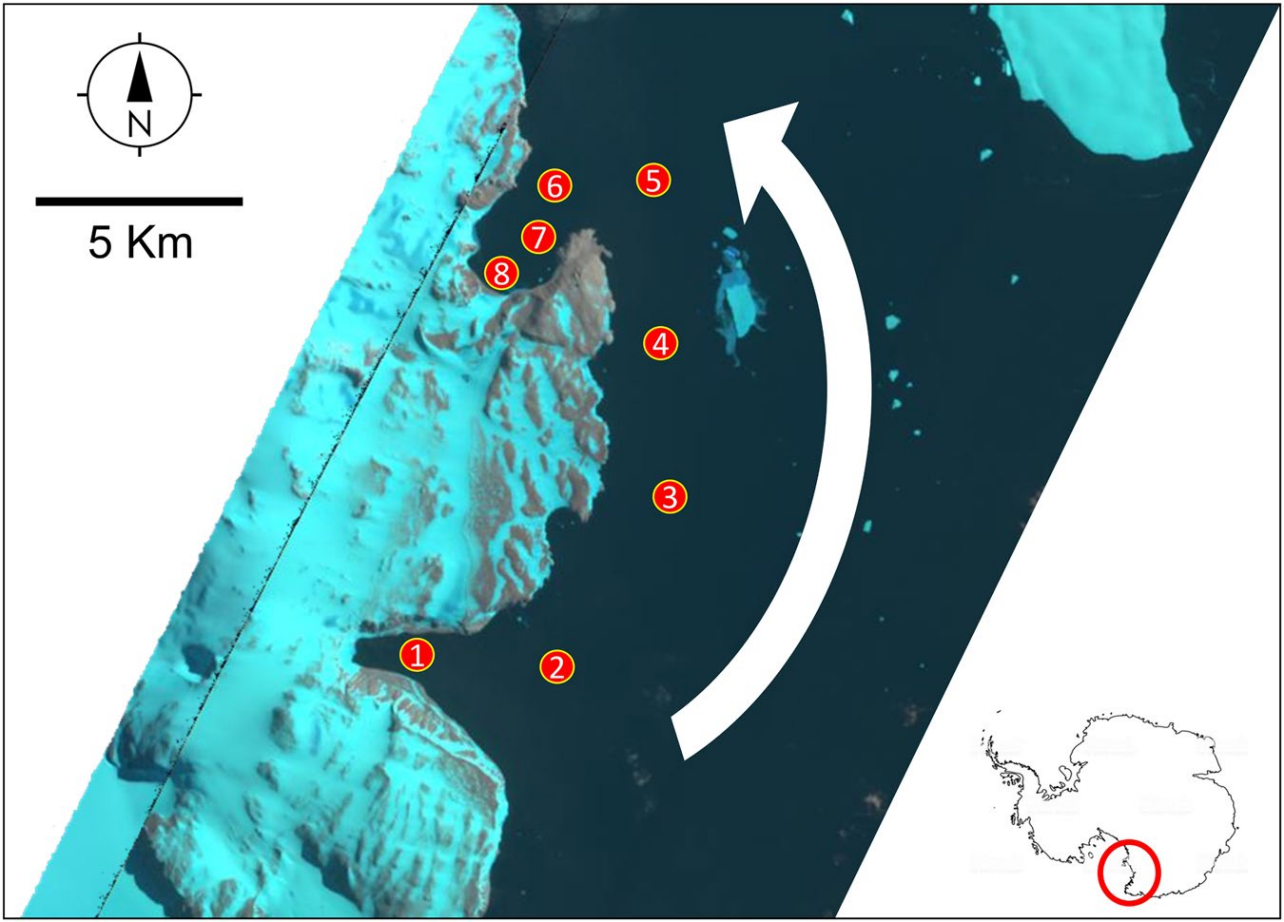
Sampling sites in Terra Nova Bay (Ross Sea). The background image of Terra Nova Bay was obtained by the Earth Observing-1 Satellite, Path: 62, Row: 113. The white arrow indicates the temporal gradient of seasonal sea-ice break up, which typically proceeds northwards, i.e. from sites 1 and 2, in the area of Adelie Cove, to sites 7 and 8, in the area of Tethys Bay. The red circle in the small map shows the position of Terra Nova Bay (Ross Sea) in Antarctica. Please refer to Table S1 for coordinates and the number of days elapsing between sea-ice break up and sampling at each site. Satellitary image of Terra Nova Bay from 03/02/2012 was obtained from https://lpdaac.usgs.gov/ maintained by the NASA EOSDIS Land Processes Distributed Active Archive Center (LP DAAC), USGS/Earth Resources Observation and Science (EROS) Center, Sioux Falls, South Dakota, 2018.

The main purpose of this study was to describe the architecture of the food web of Antarctic coastal marine communities at the kilometre scale and to determine its variations under varying conditions of coastal sea-ice persistence. It has been observed that the spatio-temporal dynamics of sea-ice cover influences basal resource inputs^8,20^, with increased availability of sympagic algae during and soon after sea-ice break up^5,18,21–24^. In accordance with optimal foraging theory, which predicts a decrease in consumer diet breadth as the per capita availability of resources increases^13,18,25^, we tested the hypothesis that food web architecture changes towards a reduction in feeding link density, and consequent web simplification, following the seasonal input of sympagic algae associated with sea-ice melting^5,18,24^. To this end, we used a “space for time” approach, sampling across multiple sites in Terra Nova Bay (TNB, Ross Sea) (Fig. 1 and Table S1), which we expected to experience varying sympagic inputs due to differences in the timing of local sea-ice break up and sea current circulation patterns^5,18,24,26,27^. Thus, based on the analysis of stable isotopes and Bayesian mixing models, the ITU-food webs were described in two adjacent areas: one where sea-ice break up occurred a few days before sampling and its possible effects on the isotopic signatures of benthic organisms were not yet detectable^18^ (Before-Breaking Diet, hereafter *BEFORE*), and the other where sea-ice break up started more than one month before sampling (reflecting the After-Breaking Diet, hereafter *AFTER*).

Comparisons were extended from web to species level, focusing on the sea urchin *Sterechinus neumayeri*, an opportunistic omnivorous feeder, which is also an important prey for abundant predator taxa such as asteroids and anemones^7,18,28,29^. *S*. *neumayeri* is among the most common benthic species in Antarctic coastal waters. It plays a key role in matter recycling and energy flows through food webs^18,28,29^, being able to feed both on vegetal and animal matter, and to vary its diet over space and time according to resource availability^18,28,29^.

## Results

### Isotopic niches

**Figure 2:**
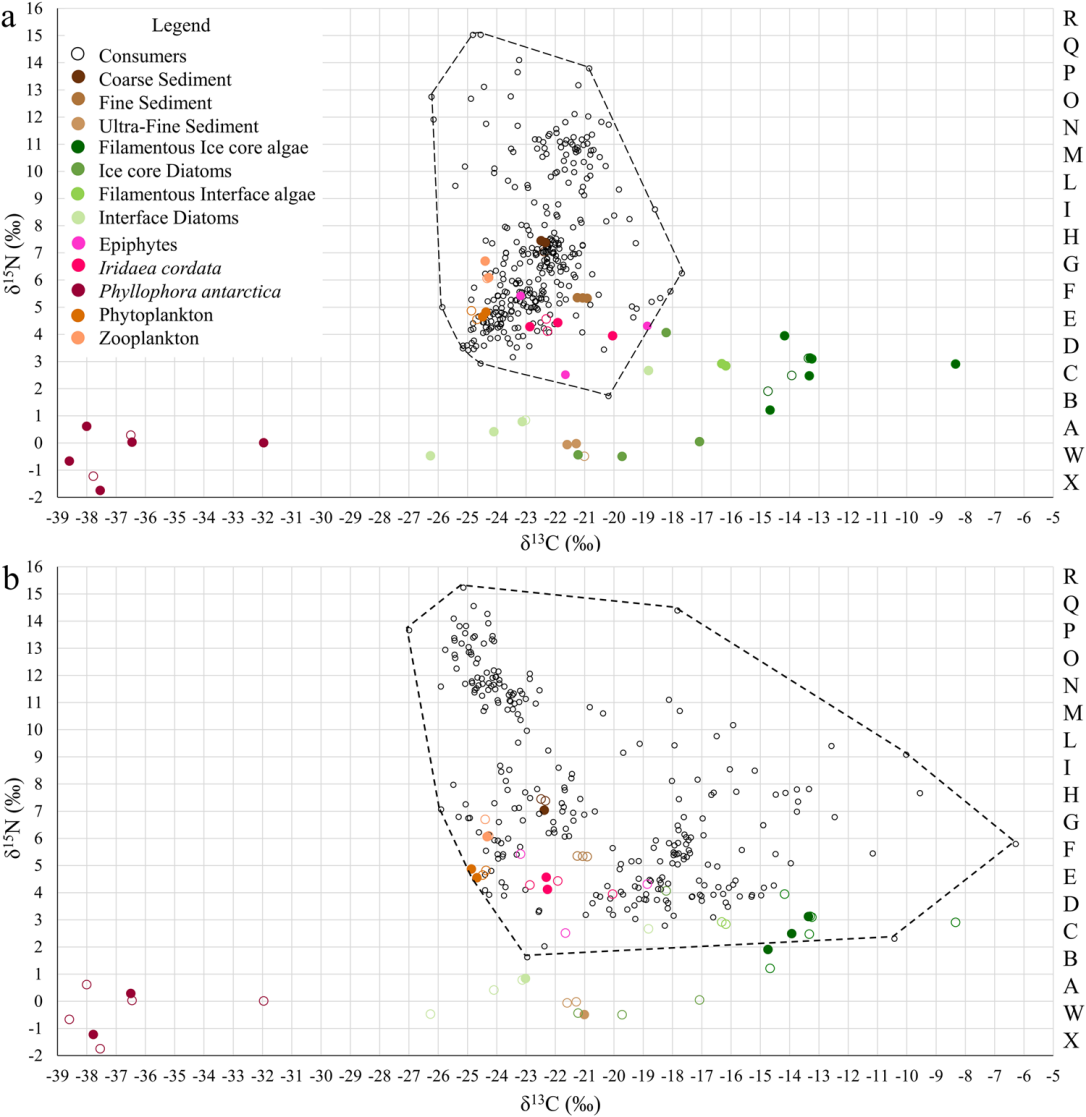
Isotopic niche biplot of BEFORE (**a**) and AFTER (**b**) communities. Each point represents a sample. The legend shows the respective colours of consumers and basal resources. Full symbols represent the basal resources of the respective study location. Empty coloured symbols represent the basal resources of the other study location. The isotopic niche space was divided into Isotopic Trophic Units (ITUs), i.e. squares having 1 × 1‰ ranges of δ^15^N and δ^13^C values, which were defined as groups of individuals occupying the same position in the δ^13^C-δ^15^N niche space. Dashed-line polygons delimit the convex hull (Total Area) of consumers.

Both the δ^13^C and δ^15^N values of the basal resources differed significantly among guilds (i.e. sympagic algae, plankton, benthic algae, epiphytes, and sediment organic matter) but not between *BEFORE* and *AFTER* areas (Fig. 2; two-way PERMANOVA, Guild: F = 56.1, p < 0.0001; Location: F = 0.50, n.s.; Interaction: F = 0.43, n.s). Specifically, sympagic algae and the red alga *Phyllophora antarctica* had the highest and lowest δ^13^C values respectively, while sediment organic matter, the red alga *Iridaea cordata* and epiphytes had intermediate δ^13^C values. The isotopic values of Adelie penguin guano were outside the isotopic spectrum of both resources and consumers (δ^13^C = −28.4 ± 0.2‰, and δ^15^N = 11.3 ± 0.6‰, n = 16) and hence were not considered for the Mixing Models.

A total of 327 and 273 individuals belonging to 86 and 41 zoobenthic taxa were respectively identified in *BEFORE* and *AFTER* sites and underwent SIA (Table S2).

**Figure 3:**
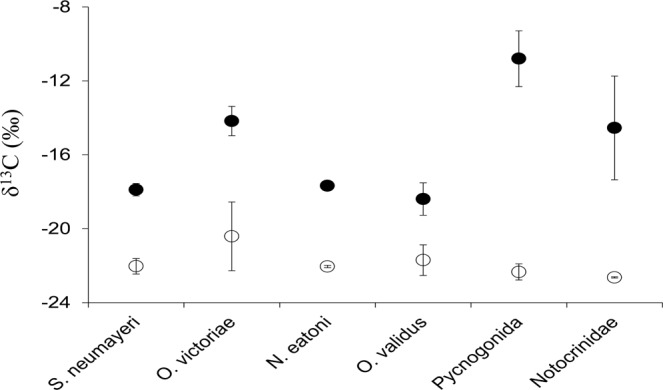
δ^13^C values of benthic organisms. Comparison between areas (i.e. BEFORE sea-ice break up, empty symbols, and AFTER sea-ice break up, black symbols) of the mean ± s.e. δ^13^C values of six generalist taxa commonly found in Antarctic benthic communities.

The mean δ^13^C values of benthic animals differed among sampling sites (One-way ANOVA, F = 72.3, p < 0.0001), being positively correlated with the number of days since sea-ice break up (n = 8, y = 0.09 × −23.05, r = 0.94, p = 0.0004, permutation p = 0.0033, 95% bootstrapped confidence interval on intercept = −23.53, −22.47). Mixture model clustering grouped the *BEFORE* and *AFTER* sites into two clearly distinct clusters (sites 5–8 and sites 1–4) with more variable and higher average δ^13^C values in the latter than the former (Table 1 and Table S1; mixture model clustering based on the EM algorithm, Bayesian Information Criterion = −4477.5). Higher δ^13^C in *AFTER* was also observed at the taxon level (Fig. 3), with the Location factor having a dominant effect (Two-way ANOVA, Location: F = 126.8, Taxon: F = 11.3, Interaction: F = 6.3, p always < 0.0001). Specifically, consumer δ^13^C values in *AFTER* were distributed along the entire isotopic spectrum of basal resources, including values typical of sympagic algae (Fig. 2).

**Table 1:** Isotopic niche and food web metrics in BEFORE and AFTER areas in Terra Nova Bay, Ross Sea.

	Before	After
**Isotopic niche metrics**		
δ^13^C°°°	−22.3 ± 0.2‰	−19.3 ± 0.5‰
δ^15^N	8.3 ± 0.4‰	7.5 ± 0.3‰
Variance δ^13^C^§§§^	4.0‰	20.2‰
Variance δ^15^N	11.0‰	10.8‰
CR	8.5‰	20.7‰
NR	13.3‰	13.6‰
TA	80.4‰^2^	186.1‰^2^
MND°°°	4.8 ± 0.1‰	6.9 ± 0.2‰
**Food web properties** (**fraction**)		
Basal Level	0.19	0.15
Intermediate*	0.42	0.59
Top*	0.39	0.26
**Link properties** (**complexity**)		
L/S^•••^	7.5 ± 0.5	5.7 ± 0.3
Cmin***	0.18	0.11
**Links** (**fraction**) **between**		
Basal resources-consumers**	0.27	0.41
Predators-prey**	0.73	0.59
**Chain properties**		
Mean chain length	2.23	2.49
Mean trophic position^•^	4.2 ± 0.2	3.7 ± 0.2
Degree of Omnivory	0.77	0.77
Degree of Intraguild Predation**	0.42	0.23
Fraction of competitors^••^	0.40 ± 0.01	0.28 ± 0.01
Robustness to primary extinctions*	0.45	0.38

CR: Carbon Range, NR: Nitrogen Range, TA: Total Area, MND: Mean Neighbour Distance, L: number of feeding links and S: number of nodes (i.e. Isotopic Trophic Units, ITUs, in the food web). Mean values are presented as mean ± standard error. Superscript symbols indicate a significant difference between locations (*χ^2^ test; ^•^Mann-Whitney test; °t-test for unequal variances, ^§^F test for variances). One, two and three superscript symbols indicate p values of <0.05, <0.01 and <0.001 respectively.

As regards δ^15^N values, although no differences were found in mean and variance, distribution varied significantly, being normally distributed in *BEFORE* and right-skewed in *AFTER* (Shapiro-Wilk test, p-normal > 0.05; Skewness = 0.07, kurtosis = −1.05 vs. p-normal < 0.05; Skewness = 0.47, kurtosis = −0.61).

The community occupied a larger isotopic niche space (TA) and individuals were isotopically more widely distributed (MND) in *AFTER* than in *BEFORE* (Table 1 and Fig. 2). Therefore, a much larger number of ITUs, together with a lower average number of taxa/individuals per ITU, was found in the former than the latter: 108 ITUs (1.54 ± 0.11 taxa and 3.01 ± 0.33 individuals per ITU) vs. 83 ITUs (2.94 ± 0.39 taxa and 4.88 ± 0.77 individuals per ITU) (Figs 2 and S1).

### Food web structures

In both food webs, the isotopic distances between ITUs significantly reflected differences in the composition of their diets (Mantel test, *BEFORE*: R = 0.56, p < 0.00001; *AFTER*: R = 0.47, p < 0.00001).

**Figure 4:**
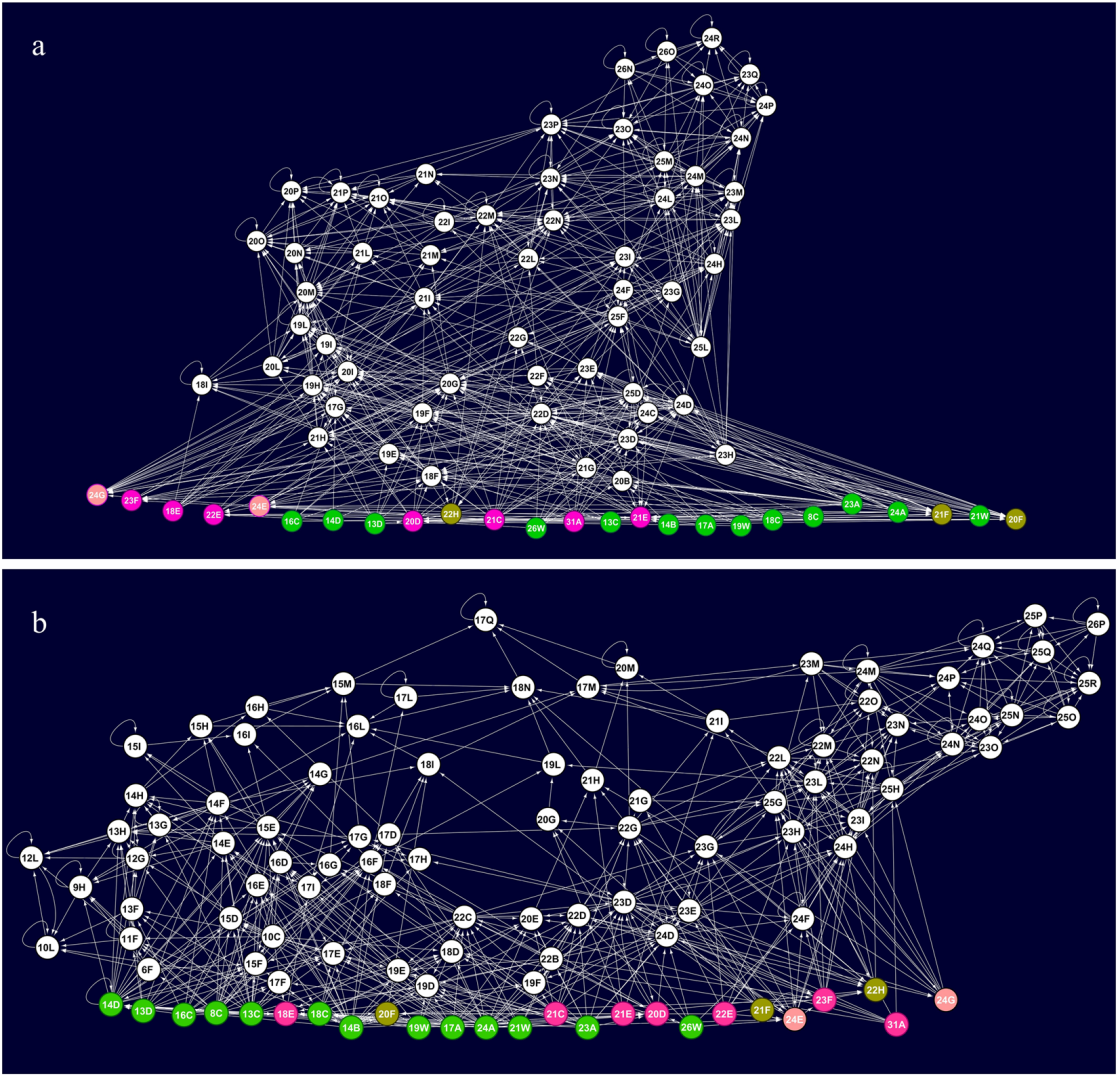
Benthic food web structure in Terra Nova Bay, Antarctica: BEFORE (**a**) and AFTER (**b**) sea-ice break up. Each node represents one Isotopic Trophic Unit (ITU) in the community. Isotopic Trophic Units (ITUs) i.e. squares having 1 × 1‰ ranges of δ^15^N and δ^13^C values, were defined as groups of individuals occupying the same position in the δ^13^C-δ^15^N niche space. Arrows point from a resource to its consumer. Nodes containing basal food sources are highlighted in different colours: green = sympagic algae, brown = organic matter in sediments, pink = plankton, violet = macroalgae. Letters and numbers identify each node as displayed in Fig. 2 and Fig. S1. The food web graphs were developed using Cytoscape software. For the list of taxa in each ITU please refer to Table S2.

Several food webs characteristics differed between areas. Specifically, the food web was more complex in *BEFORE* than in *AFTER*, as denoted by the higher feeding linkage density, which was associated with higher minimum connectance (Cmin), intraguild predation and competition between ITUs (Table 1 and Fig. 4).

**Figure 5:**
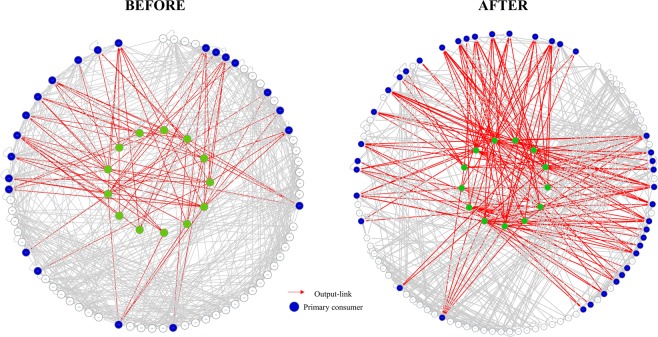
Sympagic algae-based food webs in BEFORE and AFTER areas. Each node represents one Isotopic Trophic Unit and each edge represents one trophic link. Isotopic Trophic Units (ITUs), i.e. squares having 1 × 1‰ ranges of δ^15^N and δ^13^C values, were defined as groups of individuals occupying the same position in the δ^13^C-δ^15^N niche space. Nodes at the centre of each network represent the ITUs containing sympagic algae. Peripheral nodes represent direct (blue) and indirect consumers (white, i.e. included in the source network but not feeding directly on nodes at the centre). Red edges represent direct links between basal resources and consumers, grey edges the other links in the source network. The food webs were developed using Cytoscape software.

The distribution of ITUs and feeding links across trophic levels also differed between areas (Table 1). While the degree of omnivory was similar and high in both food webs, only 27% of total links were with basal food sources in *BEFORE* (Table 1) compared to 41% in *AFTER*, where the mean trophic position of ITUs was correspondingly lower (Table 1), largely due to increased assimilation of sympagic algae (12% vs. 28% of total links in the web, χ^2^ test, χ^2^ = 48.9, p < 0.0001; Fig. 5). Specifically, in *BEFORE* sympagic algae were consumed by only 18% of individuals, including those of *Ophionotus victoriae* (Echinoidea) and *Echinopsolus mollis* (Holothuroidea), which were found to assimilate this food source in both areas. In *AFTER*, sympagic algae were consumed by 41% of individuals, including those of Pycnogonida and *Staurocucumis turqueti* (Holothuroidea), which were not found to assimilate this food source in *BEFORE*.

Lower linkage density implies lower robustness of the web to bottom-up biodiversity loss (*sensu* Dunne *et al*.^30^) (Table 1). The percentage of ITUs secondarily lost following simulated primary ITU extinctions in *BEFORE* was half what it was in *AFTER* (18% vs. 37%, χ^2^ test, χ^2^ = 8.3, p < 0.01). However, both food webs were more vulnerable to top-down than bottom-up propagation of disturbance, as indicated by the direct correlation between δ^15^N and the closeness centrality of ITUs (*BEFORE*: r = 0.68, p < 0.0001; *AFTER*: r = 0.45, p < 0.0001), which quantifies their topological proximity in the web and, thus, the potential for disturbance propagation. This implies that taxa at higher trophic levels, such as the common predators *Trematomus bernacchii*, *T*. *hansoni* and *Crionodraco hamatus*, had a higher potential to propagate disturbance than other organisms. On average, the closeness centrality of ITUs was higher in *BEFORE* than in *AFTER* (0.55 ± 0.02 vs. 0.49 ± 0.02; Mann-Whitney, U = 3537.5, p = 0.01). The 10 most central ITUs in the *BEFORE and AFTER* food webs included 21 and 9 animal taxa respectively, having in common, as primary consumers, the demosponge *Haliclona dancoi* and the bivalve *Adamussium colbecki* and, as predators, the gastropod *Neobuccinum eatoni* and the fish *T*. *bernacchii*. In *AFTER*, the 10 most central ITUs included three basal resources (filamentous sympagic algae, sympagic diatoms and epiphytes).

### Diet of the key species *Sterechinus neumayeri*

#### Comparisons between areas

As observed, on average, at the food web level, the sea urchin *S*. *neumayeri* displayed lower δ^13^C values and higher δ^15^N values in *BEFORE* than in *AFTER* (δ^13^C: −21.8‰ ± 0.49 vs. −17.55‰ ± 0.50; δ^15^N: 5.19‰ ± 0.50 vs. 4.14‰ ± 0.19, Man Whitney test, p < 0.05 for both), and its omnivorous diet varied remarkably between areas. Specifically, the ITUs occupied by the sea urchin had a lower proportion of links with basal resources in the former than in the latter (43% vs. 79%; χ^2^ test, χ^2^ = 67.2, p < 0.0001), where the consumption of sympagic algae was higher (accounting for 32% and 64% of the animal’s trophic links respectively; χ^2^ test, χ^2^ = 23.2, p < 0.0001). Accordingly, its trophic position was significantly lower (TP_BEFORE_ = 2.97 ± 0.23 and TP_AFTER_ = 2.28 ± 0.07; t-test, t = 3.3, p < 0.01). Some other food resources were used in both areas (e.g. epiphytes, ascidians and the macroalga *Phyllophora antarctica)*, whereas others were consumed only in *BEFORE* (e.g. polychaetes and carcasses of the abundant sea-star *Odontaster validus)* (Fig. 4 and Table S2). On the other hand, the description of trophic links between ITUs allowed to observe predatory links affecting *S*. *neumayeri*. As an example, specimens of *O*. *validus* and *O*. *victoriae*, which occupied ITUs 21H and 17G in BEFORE, and ITUs 16H and 13H in AFTER, fed on specimens of *S*. *neumayeri* contained in ITUs 19F and 20D (in BEFORE) and ITUs 17D and 14E (in AFTER).

### Comparisons among approaches

The diet of the sea urchin population of *AFTER* extrapolated by the mixing models from the single ITU values (individual approach) was compared with the diet determined with the two population-level approaches, which take into account all the isotopic values of *S*. *neumayeri* and the mean and standard deviations of its potential food sources. Thirteen food sources were identified and included in the diet by the individual approach (Table 2), while only 5 food sources were found by the classical population-level approach (Table 2). Although both of these approaches indicated that feeding on basal resources prevailed, in the individual approach the assimilation of animal-derived food was more pronounced (12% of the diet) than in the classical approach (3%). This difference was also found when a population-level approach based on the means and standard deviations of the 13 sources extrapolated in the individual-based diet was followed (Table 2).

**Table 2:** Diet of *Sterechinus neumayeri*.

Resources	Classical approach (Population level)	ITU approach (Individual level)	*a posteriori* approach (Population level)	References
Notocrinidae (Crinoidea)	—	5.34% ± 0.001	1.42%	^80^: reporting the congeneric *S*. *antarcticus* feeding on Crinoidea
Polyeunoa (Polichaeta)	2.33%	1.64% ± 0.002	0.30%	^7, 55, 81^
Demospongiae	—	0.31%	0.49%	^7, 29, 55^
*Echinopsolus mollis* (Holoturoidea)	—	3.3% ± 0.004	1.12%	First report by this study
*Cnemidocarpa verrucosa* (Ascidiacea)	—	0.6% ± 0.001	0.37%	^28^: reporting the congeneric *S*. *antarcticus* feeding on Ascidiacea
*Ophionotus victoriae* (Ophiuroidea)	—	0.35%	0.60%	^36^
Filamentous ice core algae	26.98%	28.74% ± 0.001	24.80%	^7, 28^
Filamentous interface algae	—	7.46% ± 0.003	2.13%	^7, 28^
Ice core Diatoms	27.27%	13.05% ± 0.001	34.19%	^7, 28, 55^
Interface Diatoms	—	25.79% ± 0.001	6.63%	^7, 28, 55^
Epiphytic diatoms	—	6.79% ± 0.003	0.64%	^28^
Ultra-fine Sediment	33.54%	4.79% ± 0.001	26.70%	^7, 28, 55^
*Phyllophora antarctica* (Florideophyceae)	—	1.84% ± 0.003	0.62%	^28, 55^
*Iridaea cordata* (Florideophyceae)	9.88%	—	—	^7, 28, 55, 81^

Proportional contribution of food sources to the diet of *Sterechinus neumayeri* in AFTER (i.e. after sea-ice break up) obtained from mixing models using various procedures for food source selection. In the ITU approach, the contribution of each food source is reported as the mean (±S.E.) contribution of that source to the ITUs of *S*. *neumayeri* (for details please refer to the methods section). The classical approach refers to the proportional contributions obtained at the population level starting from the isotopic mean and S.D. of potential prey taxa and resources. In the *a posteriori* approach, the mixing model at population level was applied using the 13 food sources identified with the ITU approach. The References column lists bibliographical references (as numbers) to previous observations of selected food sources, either alive or as carrion.

## Discussion

This study examines for the first time the food web structure of Antarctic benthic communities in medium-deep waters, where most of the Antarctic biodiversity lies^8,31^ and where the spatio-temporal sea-ice dynamics are expected to affect basal resource inputs^7,18,24,32–34^. In these highly dynamic environments, the isotopic characterisation of single individuals was crucial for describing the functional organisation of the community, including the topology of the feeding network that underlies nutrient and energy transfer across trophic levels^12,13,15,17^. Indeed, the observed complex trophic structures, which were consistent with what is expected in Antarctic benthic communities where omnivory, trophic generalism, necrophagy and intraguild predation are frequent^7,35,36^, were supported by variation in resource use between individuals within local species populations. Although it is difficult to disentangle these complex interactions, neglecting trophic variability among individuals is thus a potential source of bias in depicting food webs and energy pathways, leading to oversimplification^37^. Furthermore, the presence of a large number of potential food sources, including some with strong isotopic overlap, can reduce the discriminatory power of the mixing model and thus the effectiveness of Stable Isotope Analysis in food web reconstruction^38^. From this perspective, the concept of ‘trophospecies’ based on SIA applied at the individual level (giving rise to the ITUs in this study) represented a useful approach to the reconstruction of Antarctic food webs.

Although this approach does not allow to directly depict trophic links between taxa, it allows (i) to extrapolate trophic links between taxa starting from the information provided by the ITU-based food web, as we demonstrated for the sea urchin *S*. *neumayeri*, and (ii) to better compare food webs characterised by inconsistent taxonomic resolution^37^, provided that the isotopic analysis of specimens is performed at the individual level. On the other hand, our approach may be sensitive to undersampling or differences in sampling effort, when communities are compared, given that a low sampling effort can fail to consider the whole isotopic variability of populations. In our study, the similar high number of individuals sampled within each area gives us confidence on the results from the comparison between the two food webs. Furthermore, unambiguous extrapolation of trophic links between taxa may be difficult when a single ITU contains a high number of taxa for which previous information on diet is scarce. This risk is expected to increase with increasing size of ITUs, while it is avoided by selecting an isotopic grid of 1 × 1‰ in size as in our case (Fig. S2).

ITUs of 1 × 1‰ in size represent minimum isotopic niche units that made it possible to compare two communities where consumers showed a different variability in their isotopic signatures. It also allowed obtaining food webs composed by nodes representative of an equal portion of the community niche space, while excluding ITUs with an extremely large and variable number of individuals (Fig. S2). Since 1‰ is among the highest carbon isotopic discrimination factors expected for aquatic animals^39^, differences exceeding 1‰ on the δ^13^C axis can be considered indicative of differences in diet between individuals. This is corroborated by the strong linear correlation observed between the similarity in feeding links and the isotopic distance between ITUs. While the nitrogen isotopic discrimination factor is generally wider (~2.3‰ and ~3.4‰ for invertebrates and fishes respectively^39^), consideration of a range of 1‰ on the δ^15^N axis allowed to split individuals belonging to omnivorous populations into different trophic units, thus preserving information on food web complexity and vertical distribution of individuals along food chains.

Consideration of the mean and variance of the single ITUs’ isotopic signatures, defined by a common restricted δ-space, together with the multiple steps adopted for the selection of resources to be used in isotopic mixing models, allowed us to overcome the limits of Bayesian mixing when applied to complex communities^38,40^. Following our approach, it was thus possible to describe in unprecedented detail the food web structure of these biodiverse communities as well as the diet of single species, such as the omnivorous key species *S*. *neumayeri*, starting from individual feeding preferences without *a priori* reducing information on population diets. Finally, spatial comparisons, supported by cluster analysis and the correlation between benthic isotopic carbon signatures and sea-ice persistence, emphasised the importance of sea-ice algae input, which is affected by sea-ice extent and/or duration^5,18^, in structuring medium-depth Antarctic benthic food webs. The marked spatial effect of sea-ice break up on the isotopic signatures of organisms and food web structure suggest that the potential redistribution of sympagic material across sites due to sea currents was not relevant. Furthermore, the isotopic signatures excluded any effect of the guano of Adélie penguins, as organic input, on the isotopic composition of basal resources and benthic organisms, either before or after sea-ice break up.

Our results showed that the input of sympagic food sources following coastal sea-ice melting affected the foraging choices of benthic individuals. In turn, this reshaped the entire food web by modifying its architecture and functioning. Specifically, following the seasonal sympagic inputs, the food web was characterised by a relaxed structure with the δ^13^C values of consumers distributed along the entire isotopic spectrum of basal resources, while the mean δ^13^C shifted towards the values of sympagic algae^5,18^. The food web appeared less complex, having a higher number of trophospecies and trophic diversity (*sensu* Layman *et al*.^16^) but lower linkage density, than when the ice-coverage persisted and sea-ice algae were poorly assimilated in the food web. Before the sea-ice break up, the δ^13^C values of individuals were highly overlapping and their narrow distribution was centred on the isotopic values of benthic food sources. Indeed, Calizza *et al*.^18^ and Pusceddu *et al*.^32,41^ demonstrated that in the study area sympagic algae become available to the benthos during and soon after the seasonal sea-ice break up (see also Wing *et al*.^5^, Leu *et al*.^24^). It has also been noted that such sympagic inputs can reach great depth in just a few days through vertical deposition^21,42^. Since the isotopic analysis of soft tissues in organisms sampled soon after sea-ice break up (as in *BEFORE* conditions) describes their diet preceding the seasonal input of sympagic algae^7,18^, while it includes sympagic algae assimilation where sea-ice breaks up earlier (as in *AFTER* conditions), the differences in benthic diets and the consequent structural changes in the food web are clearly related to the different resource inputs.

Although rarely considered in food web descriptions, seasonal fluctuations in the availability of basal resources are directly reflected in the structure of the food web, given the trophic plasticity of its constituent species^43^. Here, in response to these changes, consumers specialised on the abundant basal resources after their seasonal release. This is in agreement with what has been observed spatially^5,7^ and temporally^18,43^ in some polar species. It is also consistent with what is expected from the foraging optimisation theory^44,45^ and what has been observed at the food web level in aquatic communities at lower latitudes^13,43,46^. Increased selectivity with regard to resources, coupled with decreased intraguild predation and competition, under conditions of increased resource availability can result in greater efficiency in terms of energy use by consumers and its delivery to top predators^25,47,48^, supporting the summer reproductive season^49^. In contrast, greater trophic generalism, as denoted by the higher linkage density observed before sea-ice break up, makes consumers more resistant to temporal/local extinction or reduction in single resources^12,13,30^, an advantage during the long and severely resource-limiting Antarctic winter and early spring. It is thus evident that by modulating food inputs, the seasonal growth and decline of sea ice is an important process in the persistence of marine Antarctic communities. It dynamically influences species interactions and food web structure, which in turn are both strictly related to community stability and resilience to perturbation^45,50,51^.

Despite the continuing difficulties in providing a solid taxonomic basis for Antarctic biodiversity, the efforts made here to identify specimens at the lowest possible taxonomic level yielded an unprecedented dataset, increasing our knowledge of the autoecological features of a large number of species and also enabling the description of new ones^52^. We found the sponge *Haliclona dancoi* and the bivalve *Adamussium colbecki*, the gastropod *Neobuccinum eatoni* and the fish *Trematotus bernacchii* among the 10 most central ITUs at both locations, while sympagic algae were found among the 10 most central ITUs only after sea-ice melting. We observed that topological centrality increased with trophic level, up to the fish that can be part of the diet of higher Antarctic predators, such as the tootfish *Dissosticus mawsoni* and its predator, the weddel seal^53,54^. This indicates that higher-level benthic consumers play a key role in the Antarctic food web, and suggests that the inclusion of top-predators in future research may represent a useful step to depict benthic-pelagic trophic coupling in Antarctic coastal ecosystems. In this context, identifying topological keystone populations that occupy central positions in the food web, either as a whole or some ITUs only, may help to identifying specific targets for management actions aimed at preserving food web stability.

Changes at the food web level following sympagic inputs reflected individual diet changes observed at the species population level. Here, we focused on the key species *Sterechinus neumayeri*. Among the most common benthic species in Antarctic coastal waters, the sea-urchin *S*. *neumayeri* is an omnivorous and opportunistic grazer and scavenger that plays a key role in Antarctic community structure and energy and matter flows^18,28,29^. It is known that *S*. *neumayeri* opportunistically feeds on debris and animal prey, live or as carrion, during the less productive season, but adopts a predominantly herbivorous diet, feeding mainly on sympagic algae when these are available^7,18,36,55^. As observed for the community as a whole, the isotopic values of *S*. *neumayeri* indicated increased assimilation of sympagic algae and a lower trophic position (due to decreased assimilation of animal-derived food) where sea-ice had already broken up, coupled with a decreased mean number of food sources. Despite the species’ acknowledged omnivory and marked trophic plasticity, the ITU-based approach was effective in describing its diet with a high level of detail. Specifically, the ITU-based food web highlighted the presence of resource types that are known to be consumed (live or as carrion) by *S*. *neumayeri* (see references reported in Table 2). Previously known food sources included Demospongiae, Notocrinidae and sympagic algae growing at the interface between sea-ice and water, which could not be detected by the classic population mixing model approach. In contrast, high-resolution local diets and feeding links can be extrapolated for the sea urchin and any other species in the community from the ITU-based food web. Indeed, this method made it possible to consider the entire spectrum of resources, without pooling them by mean isotopic similarity as in the classic method^38^, which also gives more weight to central values than marginal ones. In the case of the sea urchin, for example, the ITU approach highlighted the use (by some specimens) of the alga *Phyllophora antarctica*, which was characterised by markedly lower C isotopic values and would thus have been excluded by the classic population approach. Such approach also detected the consumption of *Echinpsolus mollis* (Holoturoidea), which was not reported before, and *Cnemidocarpa verrucosa* (Ascidiacea), which was reported to be consumed by *Sterechinus antarcticus* but not by *S*. *neumayeri*^28^. In parallel, the detailed ITU-food web description allowed to observe predatory links affecting the sea urchin, and to trace it back to its predators, as the sea star *Odontaster validus* and the ophiuroid *Ophionotus victoriae*.

In conclusion, our approach revealed the development of Antarctic organisms’ food strategies over time, which made it possible to scale the effects of seasonal resource input from the individual to the whole food web level^37^. Accordingly, it was shown that after sea ice break up, sympagic algae were assimilated by a greater number of benthic consumers in medium-depth waters, contributing to about one third of the links in the food web as a whole. The release of sympagic algae associated with seasonal sea-ice dynamics promoted foraging optimisation by benthic consumers, which specialised on a restricted number of resources, drastically modifying the architecture of coastal Antarctic biodiversity. This implies that changes in the sympagic compartment could significantly affect medium-depth benthic food web structure and functioning.

In the Southern Ocean, sympagic algae blooms can differ considerably from phytoplankton blooms on both spatial and temporal scales^23^. In parallel, the space-time sea-ice variations expected under current global climate change scenarios^56,57^ suggest that changes in sea-ice dynamics will modify the quantity and timing of resource availability to the benthic food web^23,24^. Thus, a potential phenological mismatch between resource availability and consumer demand due to climate change may produce unprecedented pressures (both bottom-up and top-down), threatening the persistence of Antarctic biodiversity^34^.

As concluding remarks, we can affirm that the ITU approach improved our understanding of trophic energy pathways along food chains in complex and biodiverse food webs. This approach should be applicable to any other habitat, starting from the individual isotopic characterisation of organisms. Notably, we presented unprecedented evidence that Antarctic food web architecture and metrics change predictably towards web simplification in accordance with optimal foraging theory, which expects a decrease in consumer diet breadth as the per capita availability of resources increases^13,25,45,46^. This improves our ability to model the expected effects of climate change on Antarctic biodiversity structure and functioning, as well as enabling comparison of future observations with present baseline conditions. In this sense, field experiments performed in areas naturally subject to marked seasonal sea-ice dynamics (as in the exceptional natural laboratory of Terra Nova Bay in the Ross Sea) represent a unique opportunity to make predictions regarding the ecological responses of communities in other polar areas where sea-ice persistence and resource inputs will be affected in the near future.

## Materials and Methods

### Ethics statement

All experimental protocols and methods were agreed with the PNRA (Italian National Antarctic Research Program), which also issued permits to collect samples in the study area on behalf of the Italian Ministry of Foreign Affairs. Permits were issued in compliance with the “Protocol on Environmental Protection to the Antarctic Treaty”, Annex II, art.3.

### Sampling area and samples collection

The study was carried out at various sites in Terra Nova Bay (TNB, Ross Sea) (Fig. 1 and Table S1). Samples were collected during PNRA (Italian National Antarctic Research Program) expeditions XXVII (mid-late January 2012) and XXVIII (mid-late January 2013). The Ross Sea is characterised by strong seasonality in sea-ice cover and primary productivity, which produces marked spatio-temporal variations in the availability of food^5,7,24,58^. The same seasonality is found in TNB^18,27,32,59^. Sampling was carried out along a coastal tract located between Adelie Cove (Southward) and Tethys Bay (Northward), nearly 15 km apart (Fig. 1). Along this tract, seasonal sea-ice break up typically proceeds northwards, implying that sea-ice coverage persists longer in the area of Tethys Bay. In order to quantify the number of days elapsing between sea-ice break up and sampling at each site, ground observations were coupled with satellite images (Earth Observing Satellite-1 and Landsat 7, target paths: 62–63, target row: 113). Sea ice break up occurred 57 days before sampling at sites 1 and 2 in the area of Adelie Cove, when sea-ice in the interior of the bay also showed evident cracking, and 62 and 36 days before sampling at sites 3 and 4 respectively. Sites 5–8, in the area of Tethys Bay, were characterised by longer seasonal sea-ice persistence and were sampled between five and nine days after sea-ice break up (Table S1). A cluster analysis (see Data analysis for details) grouped the individuals from different sites in two clusters: the first group (“*AFTER*”) included sites 1,2,3 and 4, while the second group (“*BEFORE*”) included sites 5, 6, 7 and 8 (Table S1). The dominant surface current circulation proceeds northwards in TNB, being affected by coastal morphology and sea-ice extent^26^. Sites 5-8 in the area of Tethys Bay were thus expected to be less affected by the dominant surface current than the others.

Adelie Cove is V-shaped. Tethys Bay in contrast is a small bay located further North along the coast of TNB. A colony of Adélie penguins (*Pygoscelis adeliae*) inhabits the inner part of Adelie Cove. The colony has around 11,200 breeding pairs, considered to be part of a metapopulation that includes colonies located at Inexpressible Island and Edmonson Point^60^ (in the southernmost and northernmost parts of our study area respectively). Foraging trips in this species can extend for more than 100 km^61^, and foraging penguins are also observed in Tethys Bay, both before and after sea-ice break up. Thus, given the potential flow of organic material from penguins to coastal waters through inputs of guano, fresh guano samples were collected at the rockery and considered for isotopic analyses. Further details of the study area can be found in Faranda *et al*.^6^ and Norkko *et al*.^7^. Benthic invertebrate sampling was carried out by dredging at depths ranging from 21 m to 240 m (Table S1). Fish samples were collected by net and line in the areas situated between sites 5 and 7 (samples assigned to the *BEFORE* food web) and between sites 1 and 3 (samples assigned to the *AFTER* food web). A few fish samples were also coincidentally collected while dredging for invertebrate sampling. Samples of detritus (organic matter in coarse, fine and ultra-fine sediments), macroalgae (*Iridaea cordata* and *Phyllophora antarctica*) and associated epiphytes were also collected by dredging in the areas of Tethys Bay and Adelie Cove. Similarly, sympagic algae (diatoms and filamentous algal aggregates, referred to as “filamentous algae”, contained within the ice core or at the ice-water interface) were sampled by coring the ice-pack before sea-ice break up. Plankton was collected by sampling the whole water column to a depth of 100 m with a plankton net (20-µm mesh size). Since the bulk sample was composed almost exclusively of copepods, zooplankton was carefully separated from the rest by hand under a stereoscope. To obtain the phytoplankton, the remaining sample was filtered at 100 μm and collected on pre-combusted Whatmann GF/F filters.

All individual samples used for Stable Isotopes Analysis (SIA) were identified to the finest possible level of taxonomic resolution (ideally to species level). Most Mollusca, and all Porifera, Arthropoda and Chordata were determined by morphology (the complete sponge dataset from Terra Nova Bay is available in Ghiglione *et al*.^62^). The other metazoans were determined by integrating the preliminary morphological identification with a DNA-barcoding approach. For part of the Mollusca, Echinodermata and Annelida (Polychaeta), partial cytochrome c oxidase subunit I (COI) sequences were produced at the Canadian Centre for DNA Barcoding (University of Guelph, Ontario, Canada) with LCOech1aF1/HCO2198 primers^63^. For Cnidaria, partial COI sequences were produced with COII8068F/COIOCTR primers^64^, and Internal Transcribed Spacer 2 (ITS2) sequences were produced with 5.8S-436/28S-663 and ITS2-3d/ITS2-4r primers^65,66^ at the Molecular Systematics lab, Dept. BBCD, Sapienza University of Rome. DNA-Barcodes were compared with the BLAST algorithm against the GenBank database (www.ncbi.nlm.nih.gov/nuccore), and within the BOLD Barcode Index Number system against the BOLD database (www.boldsystems.org), where there is also a large, still unpublished DNA-Barcode database of the Italian National Antarctic Museum (MNA) under the project BAMBi (Barcoding of Antarctic Marine Biodiversity, PNRA 2010/A1.10 project). Accession numbers are provided in the supplementary material, Annex 1.

### Laboratory procedures and stable isotope analysis

Specimens were individually stored at −80 °C and freeze-dried for 24 hours. Before the analysis, each specimen was pulverised in a ball mill (Mini-Mill Fritsh Pulverisette 23: Fritsh Instruments, Idar-Oberstein, Germany). Where necessary, in order to eliminate inorganic carbon, samples were pre-acidified using 1M HCl according to the drop-by-drop method^67^. To prevent acidification interfering with Nitrogen analysis, δ^15^N signatures were measured in un-acidified powders. After acidification, the sampled powders were re-dried (60 °C) for 72 hours to remove the remaining moisture. Aliquots of 0.25 ± 0.10 mg for the animals and 1.00 ± 0.10 mg for detritus, phytoplankton and benthic and sympagic algae were pressed into tin capsules for the Stable Isotopes Analysis. Samples were analysed in two replicates using an Elementar Vario Micro-Cube elemental analyser (Elementar Analysensysteme GmbH, Germany) coupled with an IsoPrime100 continuous flow mass spectrometer (Isoprime Ltd., Cheadle Hulme, UK), at the Trophic Ecology laboratory (Dept. of Environmental Biology, Sapienza University of Rome). Carbon (C) and Nitrogen (N) isotopic signatures were expressed in *δ* units (δ^13^C; δ^15^N) as parts per-thousand (‰) deviations from international standards: Vienna Pee Dee Belemnite (PDB) for C and atmospheric N_2_ for N. Isotopic ratios were computed according to the equation: δX (‰) = [(R_sample_ − R_standard_)/R_standard_] × 10^3^, where X is the Carbon or Nitrogen isotope and R is the heavy-to-light isotope ratio of the respective element (^13^C/^12^C; ^15^N/^14^N). Finally, δ^13^C values were corrected for lipid content based on the C/N ratio of each sample in accordance with Post *et al*.^68^. The internal laboratory standard used was IAEA600 Caffeine. δ^13^C and δ^15^N measurement errors were typically smaller than ±0.05‰.

### Data analysis

Mixture model clustering^69^ was applied in order to group sampling sites into different classes based on the log-transformed number of days elapsing between sea-ice break up and sampling and the δ^13^C and δ^15^N values of benthic organisms. Cluster parameters were estimated using an EM algorithm^70^. Model selection was based on the Bayesian Information Criterion^71^ (BIC), and the model assumes that each class is described by a multivariate Gaussian distribution with full covariance matrix. In order to confirm the observed grouping of sites into the two broader “locations” (i.e. *AFTER* and *BEFORE*), a two-way ANOVA testing the effect of Location, Taxon and their interaction was applied to the δ^13^C values of six generalist benthic taxa commonly found in Antarctic benthic communities, i.e. the sea urchin *Sterechinus neumayeri*, the brittle star *Ophionotus victoriae*, the gastropod *Neobuccinum eatoni*, the seastar *Odontaster validus* and specimens of Pycnogonida and Notocrinoidae.

Isotopic data of collected organisms were used to reconstruct the food webs at each location, where nodes represented isotopic trophospecies. Isotopic trophospecies are defined as groups of individuals with very similar isotopic signatures, which therefore occupy the same position in the δ^13^C-δ^15^N niche space. In this sense, the bi-dimensional isotopic space was subdivided into ITUs (Isotopic-Trophic-Units), i.e. squares having 1 × 1‰ ranges of δ^15^N and δ^13^C values, starting from the lowest δ^13^C value present in the dataset and a δ^15^N value of zero and proceeding in ascending order. The ITUs were univocally identified by a number on the δ^13^C axis and a letter on the δ^15^N axis (Fig. 2 and Fig. S1). Within the bi-dimensional isotopic space, basal resources, invertebrates and fish were identified and labelled. This was necessary for assigning the correct isotopic fractionation to vertebrates (i.e. δ^15^N: 2.1 ± 0.7‰, δ^13^C: 0.4 ± 0.2‰, based on observation in Antarctic fish^72^) and invertebrates (i.e. δ^15^N: 2.3 ± 0.4‰, and δ^13^C: 0.4 ± 0.2‰). Based on a broader literature analysis by McCutchan *et al*.^39^, these values produced meaningful mixing model outputs when applied to benthic invertebrates in our study area^7,18^. The result was a grid (in a trophochemical graph) in which each ITU could be a consumer and/or a resource of organisms (Fig. S1) whose taxonomic classification is known in detail.

The diet of each ITU was calculated by means of Bayesian Mixing Models^73^ (SIAR package in R-statistics software). The SIAR mixing models return probable resource proportion values with credibility intervals plotted at 95, 75 and 50%. This allows direct identification of the food sources in the diet of each consumer and therefore robust food web reconstruction^13,17,18,38,40,46^. For each consumer ITU, we considered a set of potential ITU food sources on the entire δ^13^C axis and within a given range on the δ^15^N axis, i.e. with values within ± 3‰ of the value of the consumer after subtracting its TEF^13,40^. Furthermore, given the high number of ITUs in each food web, the food sources were split into two subsets (A and B). Similar to white and black cells in a chessboard, the two subsets were divided so that within each subset there were no two contiguous ITUs on the δ^13^C and δ^15^N axes, while the ITUs within each subset covered the whole range of δ^13^C values observed. Based on the mixing model outputs for subsets A and B, a set of selected food sources was retained for a third mixing model. Based on the output of this model, a pool of real (i.e. likely to be assimilated) food sources was then selected in accordance with Phillips *et al*.^38^ and Costantini *et al*.^40^. At each step, a food source was retained if (i) the 95% confidence interval (CI) did not include 0, or if (ii) the 75% CI did not include 0 and its modal contribution was ≥5%, and/or (iii) it was necessary in order to obtain a mixing space encompassing the consumer^40^. Lastly, a fourth mixing model was run including only these real food sources in order to quantify the proportional contribution of each source to the diet of a consumer^40^. After the assignment of trophic links, the food web structures were reconstructed, the basal resource inputs were described and the respective food web metrics were quantified using the “foodweb” package in R and Cytoscape software^74^.

In order to validate the diet of the sea-urchin *Sterechinus neumayeri* obtained with the procedure described above, we assessed its diet and reconstructed its sub-web by a traditional approach, i.e. starting from the isotopic mean and SD of potential prey taxa and resources as selected by the literature and SIA^38,40^.

### Food web metrics

To compare the food webs of the two locations, trophic chain length was estimated from the δ^15^N range (NR) and the diversity of basal resources exploited by the community was estimated from the δ^13^C range (CR) and variance^13,15,75^. NR and CR were both calculated as the Euclidean distance between the highest and the lowest respective isotopic values (δ^15^N, δ^13^C). Total area (TA) was calculated as the convex hull encompassing the consumer data points and was expressed as isotopic area^16^ (‰^2^). Total area provides a measurement of the total niche space occupied by the community at each sampling location, while the mean isotopic distance between all ITUs in each location provides a measure of trophic-functional diversity within the community.

Trophospecies richness (S) was estimated as the number of total nodes (i.e. ITUs) present in the food web. Linkage density (L/S) was measured as the average number of feeding links (L) per ITU. Minimum Connectance (Cmin) was calculated as: C = 2L/S^2^. For each ITU, the trophic position (TP) and its closeness centrality were obtained using the foodweb package in R. The TP of a consumer takes into account the TP of the food sources it assimilates and their relative contribution to its diet, starting from TP = 1 assigned to basal food sources. The closeness centrality Cc(n) of a given node n is defined as the reciprocal of the average shortest path length. It is computed as follows: Cc(n) = 1/avg(L(n,m)), where L(n,m) is the length of the shortest path between two nodes n and m. The Cc(n) of each node is a measure, ranging between 0 and 1, of how fast information spreads from a given node to other nodes in the network^76^. Thus, it can be related to the vulnerability of a food web to perturbation propagation across nodes (ITUs in our case) and can help to identify which ITUs/species display the highest centrality in the community^77^. The importance of omnivory and intraguild predation were estimated using the foodweb package^78^ in R. Given the potential presence of different taxa within the same ITU, we refer to “intraguild predation” instead of “cannibalism”, intending that individuals contained within an ITU fed either on conspecifics and/or on individuals belonging to other taxa contained within the same ITU. The evidence for including an “intraguild” link was the same as for the selection of all the other links in the food web.

Food web robustness to ITU loss was estimated following simulated deletion of ITUs from the food web, proceeding from the most to the least connected ITU and considering all the ITUs in the food web in accordance with Calizza *et al*.^12^. The simulated deletion of an ITU is considered as a primary extinction (sensu Dunne *et al*.^30^), while a secondary extinction occurs when a non-basal ITU loses all of its prey/resource ITUs except itself. Lastly, a Mantel test was applied to assess if isotopic signatures of ITUs were predictive of the feeding links they made in the food web, considering both the identity and the relative contribution of each food source to each ITU. The Mantel test, as a permutation test for correlation between two distance or similarity matrices, allowed us to compare multivariate data with different similarity measures^79^. Here, the Euclidean distance and the Bray-Curtis dissimilarity were selected to quantify isotopic distances between ITUs and pairwise differences in feeding links respectively^72,79^.

## Supplementary information

Supplementary information

Annex 1
