# Supplementary material for: Antarctic food web architecture under varying dynamics of sea ice cover
Source: Sci Rep. 2019 Aug 28;9:12454. doi: 10.1038/s41598-019-48245-7 (PMC6713710; doi:10.1038/s41598-019-48245-7)
Supplement: Supplementary file 1 — Supplementary information [file 41598_2019_48245_MOESM1_ESM.pdf]

SUPPLEMENTARY MATERIAL

ANTARCTIC FOOD WEB ARCHITECTURE UNDER VARYING DYNAMICS OF SEA ICE COVER

Loreto Rossi[1,2], Simona Sporta Caputi[2], Edoardo Calizza[1,2,*], Giulio Careddu[2], Marco Oliverio[3], Stefano Schiaparelli[4,5], Maria Letizia Costantini[1,2].

[1] CoNISMa-Consorzio Nazionale Interuniversitario per le Scienze del Mare, Piazzale Flaminio 9, Rome (IT)

[2] Department of Environmental Biology, Sapienza University of Rome, Via dei Sardi 70, Rome (IT)

[3] Department of Biology and Biotechnologies ‘‘Charles Darwin’’, Sapienza University of Rome, Zoology–Viale dell’Università 32, Rome (IT)

[4] Department of Earth, Environmental and Life Sciences (DISTAV), University of Genoa, Corso Europa 26, Genoa (IT)

[5] Italian National Antarctic Museum (Genoa Section), University of Genoa, Viale Benedetto XV 5, Genoa (IT)

* Corresponding author: edoardo.calizza@uniroma1.it

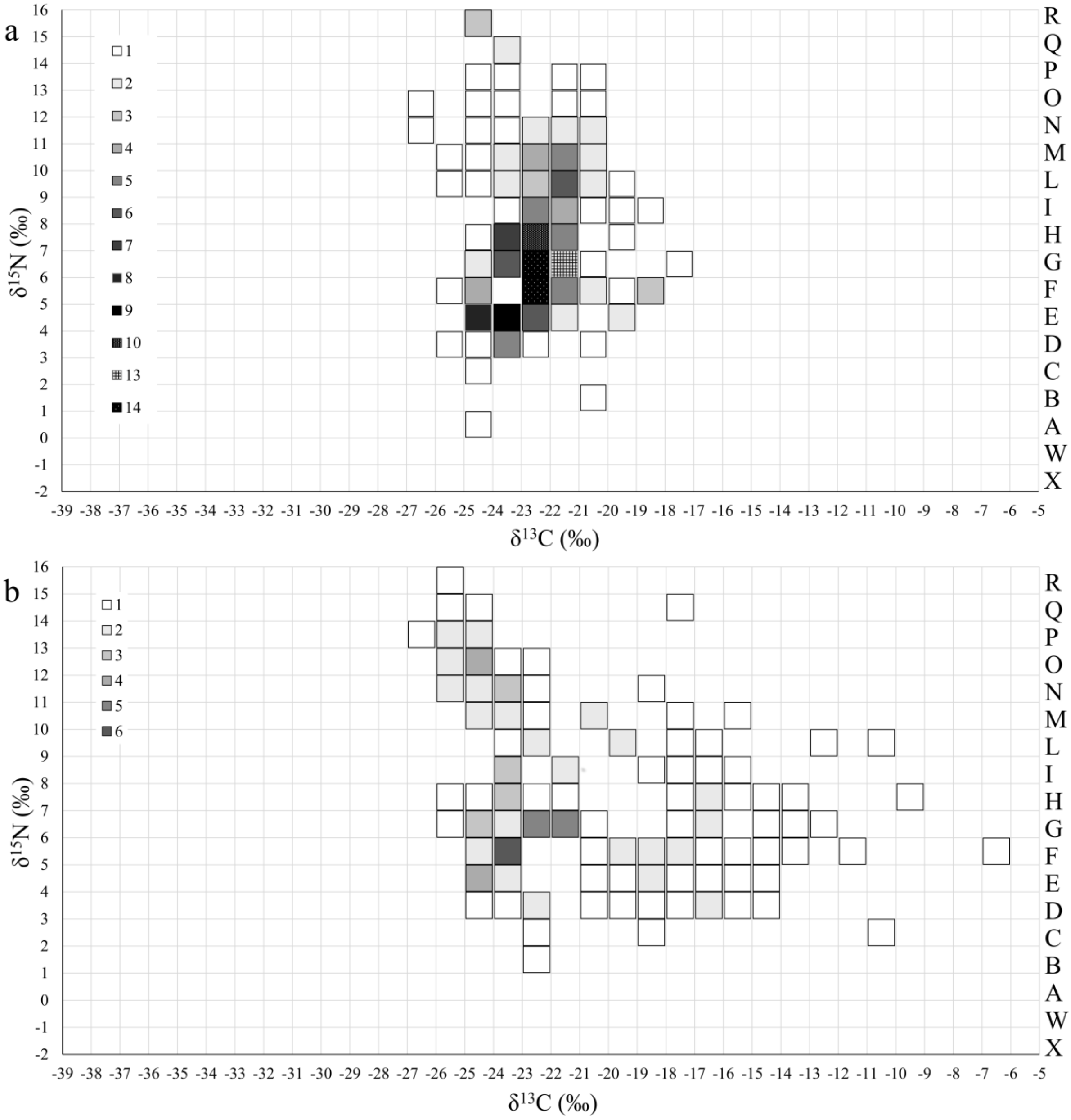

Fig. S1. Representation of Isotopic Trophic Units (ITUs) within each area (a: BEFORE sea-ice break-up; b: AFTER sea-ice break-up). Isotopic Trophic Units (ITUs) i.e. squares having 1 x 1 ‰ ranges of $\delta^{15}N$ and $\delta^{13}C$ values, were defined as groups of individuals occupying the same position in the $\delta^{13}C$-$\delta^{15}N$ niche space. Only ITUs which were actually occupied by at least one specimen are highlighted in the isotopic niche space. Different colours and textures indicate different numbers of taxonomic units included within each ITU.

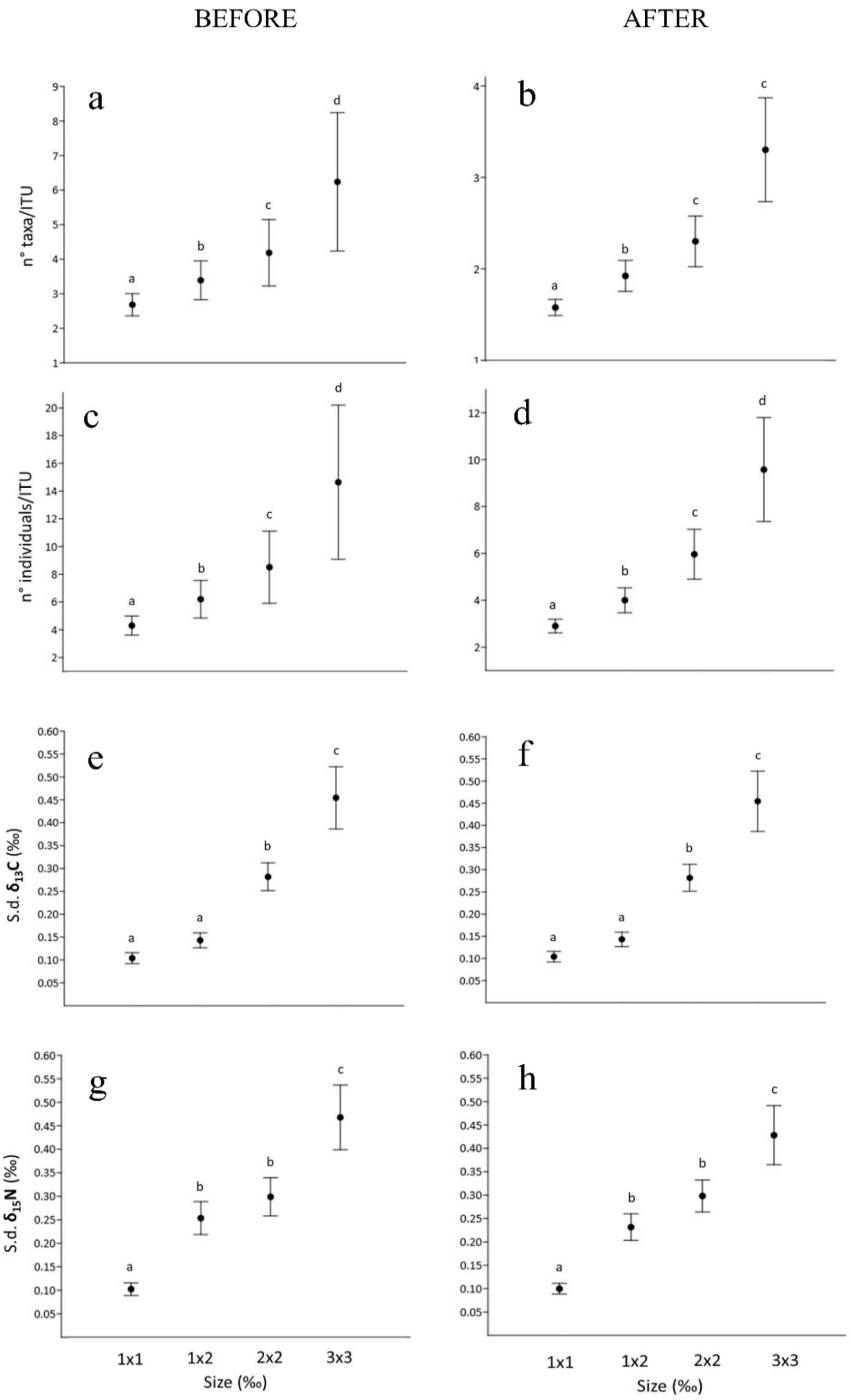

Fig. S2. Comparison among different ITU sizes. Whisker plots displaying values (mean ± standard error) of the n° of taxa per Isotopic Trophic Unit (ITU) (a-b), n° of individuals per ITU (c-d), and of the isotopic standard deviation (S.d.) of ITUs (e-h) at different ITU sizes, in BEFORE and AFTER (i.e. before and after sea-ice break up). ITU size is intended as $\delta^{13}C$ (‰) x $\delta^{15}N$ (‰). Different letters imply a significant difference between sizes (Kruskal-Wallis and Mann-Whitney pairwise comparisons, Hc value always > 14.6, p always < 0.01). For each parameter, the variance among ITUs significantly increased from size 1x1 to size 3x3 (Levene statistic for homogeneity of variance, p always < 0.001). Please note differences in scale on the vertical axis for the n° of taxa and the n° of individuals per ITU.

Table S1. Sampling sites at Terra Nova Bay.

For each site, geographical coordinates and the number of days elapsing between sea-ice break up and sampling are shown. The output of mixture model clustering, taking into account the log-transformed n° of days and $\delta^{13}C$ and $\delta^{15}N$ values of organisms, is also reported. For each site, the output includes the probability (p) and uncertainty (unc.) of assignation of organisms to either the AFTER or the BEFORE group. p = 1 implies that all organisms from a given site were assigned to the same group without uncertainty.

Site	Latitude	Longitude	Days after break up	Depth range (m)	Mixture model clustering	
					AFTER	BEFORE
1	74°46.458'S	163°57.240'E	57	61-78	p = 1, unc.= 0	0
2	74°46.642'S	164°03.171'E	57	63-140	p = 1, unc.= 0	0
3	74°44.205'S	164°10.621'E	63	190-240	p = 1, unc.= 0	0
4	74° 42.802'S	164° 08.942'E	36	40-150	p = 1, unc.= 0	0
5	74°41.271'S	164°08.547'E	7	99-208	0	p = 1, unc.= 0
6	74°41.323'S	164°03.896'E	9	21-222	0	p = 1, unc.= 0
7	74°41.839'S	164°03.549'E	5	65-170	0	p = 1, unc.= 0
8	74°42.006'S	164°02.101'E	5	42-198	0	p = 1, unc.= 0

Table S2. List of taxa present in each ITU (Isotopic Trophic Unit) in the BEFORE (a) and AFTER (b) food webs at Terra Nova Bay, Ross Sea. The Isotopic Trophic Units (ITUs), i.e. squares having 1 x 1 ‰ ranges of $\delta^{13}C$ and $\delta^{15}N$ values, were defined as groups of individuals occupying the same position in the $\delta^{13}C$-$\delta^{15}N$ niche space. The ITUs are labelled with a number and a letter to identify their positions on the $\delta^{13}C$ and $\delta^{15}N$ axis respectively.

a)

ITU-code	Taxa
8C	Filamentous ice core algae
13C	Filamentous ice core algae
13D	Filamentous ice core algae
14B	Filamentous ice core algae
14D	Filamentous ice core algae
16C	Filamentous interface algae
17A	Ice core diatoms
17G	*Ophionotus victoriae*
18C	Interface diatoms
18E	Ice core diatoms
18E	Epiphytes
18F	*Thouarella (Thouarella) pendulina*
18F	Alcyonacea
18F	*Ophidiogorgia paradoxa*
18I	*Odontaster validus*
19E	Primnoidae
19E	*Thouarella sp.*
19F	*Sterechinus neumayeri*
19H	*Lissodendoryx (lissodendoryx) flabellata*
19I	*Diplasterias brucei*
19L	Euphausia
19W	Ice core diatoms
20B	*Sterechinus neumayeri*
20D	*Iridaea cordata*
20D	*Sterechinus neumayeri*
20F	Arcturidae
20F	Fine Sediment
20F	*Parbolasia corrugatus*
20G	*Kirkpatrickia coulmani*
20I	*Ophionotus victoriae*
20L	*Odontaster validus*
20L	Polinoidae
20M	*Parania antarctica*
20M	*Trematomus bernacchii*
20N	*Trematomus bernacchii*
20N	*Trematomus pennellii*
20O	*Trematomus bernacchii*
20P	*Artedidraco orianae*
21C	Epiphytes
21E	Hydrozoa
21E	*Iridaea cordata*
21E	*Sterechinus neumayeri*
21F	Fine Sediment
21F	*Heterocucumis denticulata*
21F	*Homaxinella cf. balfourensis*
21F	*Isodictya erinacea*
21F	*Tedania (Tedaniopsis) charcoti*
21F	*Sterechinus neumayeri*
21G	*Adamussium colbecki*
21G	*Arntzia gracilis*
21G	Ascidiacea
21G	Holoturoidea
21G	*Inflatella belli*
21G	*Lissodendoryx (ectyodoryx) antarctica*
21G	*Odontaster validus*
21G	*Psolicrux coatsi*
21G	Pycnogonida
21G	*Tedania(tedaniopsis) charcoti*
21H	*Adamussium colbecki*
21H	*Inflatella belli*
21H	Holoturoidea
21H	*Odontaster validus*

21H	*Tedania sp.*
21I	*Doris kerguelenensis*
21I	*Haliclona(rhizoniera) cf. dancoi*
21I	Polinoidae
21I	*Lissodendoryx (Lissodendoryx) flabellata*
21L	*Doris kerguelenensis*
21L	Euphausiacea
21L	*Histiodraco velifer*
21L	*Lissodendoryx (Lissodendoryx) flabellata*
21L	*Trematomus bernacchii*
21M	*Doris kerguelenensis*
21M	*Lepidonotothen nudifrons*
21M	*Antarctotetilla leptoderma*
21M	*Trematomus bernacchii*
21N	*Lepidonotothen nudifrons*
21N	*Trematomus bernacchii*
21O	*Trematomus bernacchii*
21P	*Artedidraco skottsbergi*
21W	Ice core diatoms
21W	Ultra-Fine Sediment
22D	Sabellidae
22E	Ascidiacea
22E	Demospongiae
22E	*Dendrilla membranosa*
22E	Holoturoidea
22E	*Iridaea cordata*
22E	Sabellidae
22E	*Sterechinus neumayeri*
22F	*Staurocucumis turqueti*
22F	*Adamussium colbecki*
22F	Arcturidae
22F	Demospongiae
22F	*Echinopsolus mollis*
22F	*Heterocucumis denticulata*
22F	Holoturoidea
22F	*Isodictya conulosa*
22F	*Isodictya sp*
22F	*Notocrinus virilis*
22F	*Isodictya erinacea*
22F	*Pseudosuberites nudus*
22F	*Rossella cf. longstaffi*
22F	*Sterechinus neumayeri*
22G	*Staurocucumis turqueti*
22G	*Adamussium colbecki*
22G	Demospongiae
22G	*Echinopsolus mollis*
22G	*Haliclona(rhizoniera) dancoi*
22G	Holoturoidea
22G	*Isodictya conulosa*
22G	*Lissodendorix sp.1*
22G	*Mycale (Oxymycale) acerata*
22G	*Myxilla (myxilla) elongata*
22G	*Notocrinus virilis*
22G	Polichaeta
22G	*Suberites caminatus*
22H	*Adamussium colbecki*
22H	Coarse Sediment
22H	*Cucumaria georgiana*
22H	*Staurocucumis turqueti*
22H	Demospongiae
22H	*Haliclona sp.4*
22H	Holoturoidea
22H	*Myxilla (Myxilla) elongatas*
22H	*Lissodendoryx(lissodendoryx) flabellata*
22H	*Phorbas glaberrimus*
22I	*Diplasterias brucei*
22I	*Inflatella belli*
22I	*Odontaster validus*
22I	Polinoidae
22I	Pycnogonida
22L	Asteroidea
22L	*Polyeunoa laevis*
22L	*Hemigellius fimbriatus*
22M	*Neobuccinum eatoni*
22M	Polichaeta
22M	*Trematomus bernacchii*
22N	Actiniaria
22N	*Trematomus bernacchii*
23A	Interface Diatoms
23D	*Adamussium colbecki*
23D	*Echinopsolus mollis*
23D	*Heterocucumis steineni*
23D	Holoturoidea
23D	Sabellidae
23E	*Adamussium colbecki*
23E	Ascidiacea
23E	*Cucumaria georgiana*
23E	*Echinopsolus mollis*
23E	*Isodictya sp*
23E	*Limatula hodgsoni*
23E	*Psolicrux coatsi*
23E	Sabellidae
23F	*Adamussium colbecki*
23F	*Arntzia gracilis*

23F	Bryozoa	24D	*Adamussium colbecki*
23F	*Calyx arcuarius*	24E	*Adamussium colbecki*
23F	*Cucumaria georgiana*	24E	*Cnemidocarpa verrucosa*
23F	*Echinopsolus mollis*	24E	*Heterocucumis denticulata*
23F	Epiphytes	24E	Holoturoidea
23F	Holoturoidea	24E	*Limatula hodgsoni*
23F	*Isodictya erinacea*	24E	Phytoplankton
23F	*Microxina benedeni*	24E	*Psolicrux coatsi*
23F	*Psolicrux coatsi*	24E	*Trachythyone bouvetensis*
23F	Pycnogonida	24F	*Adamussium colbecki*
23F	*Rossella nuda*	24F	Holoturoidea
23F	*Staurocucumis turqueti*	24F	*Psolicrux coatsi*
23F	*Sterechinus neumayeri*	24F	*Trachythyone bouvetensis*
23G	*Cucumaria georgiana*	24G	*Staurocucumis turqueti*
23G	*Echinopsolus mollis*	24G	Holoturoidea
23G	*Haliclona(rhizoniera) cf. dancoi*	24G	Zooplankton
23G	Holoturoidea	24H	*Sterechinus neumayeri*
23G	*Kirkpatrickia coulmani*	24L	*Trematomus newnesi*
23G	*Sterechinus neumayeri*	24M	*Ophionotus victoriae*
23H	Amphipoda	24N	*Trematomus newnesi*
23H	Asteroidea	24O	*Chionodraco hamatus*
23H	*Clathria (Thalysias) flabellata*	24P	*Chionodraco hamatus*
23H	*Odontaster validus*	24R	*Chionodraco hamatus*
23H	*Sterechinus neumayeri*	24R	*Trematomus hansoni*
23H	*Trachythyone bouvetensis*	25D	*Adamussium colbecki*
23I	Demospongiae	25F	*Staurocucumis turqueti*
23L	Perknaster sp.	25L	*Odontaster validus*
23L	Polichaeta	25M	Ascidiacea
23M	*Trematomus bernacchii*	26N	*Chionodraco hamatus*
23M	*Trematomus newnesi*	26O	*Chionodraco hamatus*
23N	Parborlasia	26W	Interface diatoms
23O	*Gimnodraco acuticeps*	31A	*Phyllophora antarctica*
23P	Parborlasia	36A	*Phyllophora antarctica*
23Q	*Gimnodraco acuticeps*	37A	*Phyllophora antarctica*
24A	Interface Diatoms	37X	*Phyllophora antarctica*
24C	Ascidiacea	38W	*Phyllophora antarctica*

b)

ITU-code	Taxa		
6F	*Ophionotus victoriae*	10L	*Odontaster validus*
8C	Filamentous ice core algae	11F	*Ophionotus victoriae*
9H	Pycnogonida	12G	*Ophionotus victoriae*
10C	Notocrinidae	12L	Pyconogonida

13C	Filamentous ice core algae
13D	Filamentous ice core algae
13F	Comatulida
13G	*Ophionotus victoriae*
13H	*Ophionotus victoriae*
14B	Filamentous ice core algae
14D	Filamentous ice core algae
14D	*Sterechinus neumayeri*
14E	*Sterechinus neumayeri*
14F	*Sterechinus neumayeri*
14G	*Ophionotus victoriae*
14H	*Ophionotus victoriae*
15D	*Sterechinus neumayeri*
15E	*Sterechinus neumayeri*
15F	*Sterechinus neumayeri*
15H	*Ophionotus victoriae*
15I	*Ophionotus victoriae*
15M	*Odontaster validus*
16C	Filamentous interface algae
16D	Polyeunoa
16D	*Sterechinus neumayeri*
16E	*Sterechinus neumayeri*
16F	*Sterechinus neumayeri*
16G	Leucascidae
16G	*Aequiyoldia (Yoldia) eightsii*
16H	*Odontaster validus*
16H	Odontasteridae
16I	*Ophionotus victoriae*
16L	*Odontaster validus*
17A	Ice core diatoms
17D	*Sterechinus neumayeri*
17E	*Sterechinus neumayeri*
17F	*Aequiyoldia (Yoldia) eightsii*
17F	*Sterechinus neumayeri*
17G	*Aequiyoldia (Yoldia) eightsii*
17H	*Ophionotus victoriae*
17I	*Ophionotus victoriae*
17L	*Neobuccinum eatoni*
17M	*Neobuccinum eatoni*
17Q	Polyeunoa
18C	Interface diatoms
18C	*Sterechinus neumayeri*
18D	*Sterechinus neumayeri*
18E	Ice core diatoms
18E	Epiphytes
18E	Flabelligeridae
18E	*Sterechinus neumayeri*
18F	*Aequiyoldia (Yoldia) eightsii*
18F	Schizasteridae
18I	*Odontaster sp*
18N	*Neobuccinum eatoni*
19D	*Sterechinus neumayeri*
19E	*Sterechinus neumayeri*
19F	*Abatus sp.*
19F	*Sterechinus neumayeri*
19L	Odontasteridae
19L	Polyeunoa
19W	Ice core diatoms
20D	*Iridaea cordata*
20D	*Sterechinus neumayeri*
20E	*Sterechinus neumayeri*
20F	Fine Sediment
20F	Notocrinidae
20G	*Polyeunoa laevis*
20M	*Acodontaster sp 2*
20M	*Odontaster validus*
21C	Epiphytes
21E	*Iridaea cordata*
21F	Fine Sediment
21G	*Haliclona (Rhizoniera) cf. dancoi*
21G	*Lysianassidae*
21G	*Ophionotus victoriae*
21G	Polyeunoa
21G	*Polyeunoa laevis*
21H	*Polyeunoa laevis*
21I	Polyeunoa
21I	Polynoidae
21W	Ice core diatoms
21W	Ultra-Fine Sediment
22B	*Sterechinus neumayeri*
22C	*Sterechinus neumayeri*
22D	Demospongiae
22D	*Sterechinus neumayeri*

22E	*Iridaea cordata*
22G	*Arntzia gracilis*
22G	*Ophioplinthus gelida*
22G	*Phorbas glaberrimus*
22G	Polyeunoa
22G	*Polyeunoa laevis*
22H	Coarse Sediment
22H	*Lysianassidae*
22L	*Haliclona (Soestella) cf. chilensis*
22L	*Trematomus pennellii*
22M	*Trematomus pennellii*
22N	*Trematomus pennellii*
22O	*Trematomus pennellii*
23A	Interface diatoms
23D	*Echinopsolus mollis*
23E	*Echinopsolus mollis*
23E	*Staurocucumis turqueti*
23F	Bryozoa
23F	*Cucumaria georgiana*
23F	Epiphytes
23F	*Haliclona (Rhizoniera) cf. dancoi*
23F	Holoturoidea
23F	*Psolicrux coatsi*
23F	Styelidae
23F	*Trachythyone bouvetensis*
23G	*Lysianassidae*
23G	*Trachythyone bouvetensis*
23H	Amphipoda
23H	Holoturoidea
23H	*Lysianassidae*
23I	Amphipoda
23I	*Lysianassidae*
23I	*Trachythyone bouvetensis*
23L	Amphipoda
23M	*Trematomus bernacchii*
23M	*Trematomus pennellii*
23N	*Trematomus bernacchii*
23N	*Trematomus newnesi*
23N	*Trematomus pennellii*
23O	*Trematomus bernacchii*
24A	Interface diatoms
24D	*Cnemidocarpa verrucosa*
24E	*Cucumaria georgiana*
24E	Holoturoidea
24E	Phytoplankton
24E	*Psolicrux coatsi*
24E	*Trachythyone bouvetensis*
24F	Holoturoidea
24F	*Trachythyone bouvetensis*
24G	Amphipoda
24G	*Cnemidocarpa verrucosa*
24G	*Lysianassidae*
24G	Zooplankton
24H	Amphipoda
24M	*Trematomus bernacchii*
24M	*Trematomus newnesi*
24N	*Trematomus bernacchii*
24N	*Trematomus newnesi*
24O	*Chionodraco hamatus*
24O	*Trematomus bernacchii*
24O	*Trematomus hansoni*
24O	*Trematomus newnesi*
24P	*Chionodraco hamatus*
24P	*Trematomus hansoni*
24Q	*Trematomus hansoni*
25G	*Lysianassidae*
25H	Amphipoda
25N	*Trematomus bernacchii*
25N	*Trematomus newnesi*
25O	*Chionodraco hamatus*
25O	*Trematomus hansoni*
25P	*Chionodraco hamatus*
25P	*Trematomus hansoni*
25Q	*Trematomus hansoni*
25R	*Trematomus hansoni*
26P	*Trematomus hansoni*
26W	Interface Diatoms
31A	*Phyllophora antarctica*
36A	*Phyllophora antarctica*
37A	*Phyllophora antarctica*
37X	*Phyllophora antarctica*
38W	*Phyllophora antarctica*
